# Coupled coordination relationship and enhancement path between digital economy and essential public health services in China

**DOI:** 10.3389/fpubh.2025.1517353

**Published:** 2025-05-07

**Authors:** Kunyu Chen, Qunshan Tao, Yang Wang, Zili Ding, Rui Fu

**Affiliations:** ^1^School of Hospital Economics and Management, Anhui University of Chinese Medicine, Hefei, China; ^2^Key Laboratory of Data Science and Innovative Development of Chinese Medicine in Anhui Province Philosophy and Social, Hefei, China; ^3^Wuxi Second People's Hospital, Wuxi, China

**Keywords:** WSR methodology, essential public health services, digital economics, coupled coordination, configuration analysis

## Abstract

**Introduction:**

The coordinated development of the digital economy and essential public health services is a critical issue for advancing the Healthy China initiative and promoting health equity. However, existing policy frameworks exhibit significant shortcomings in the design of cross-system collaborative governance tools and regional adaptability, thereby constraining the implementation effectiveness of the “digital health” strategy.

**Methods:**

This study constructs an evaluation index system for the digital economy and essential public health services based on panel data from 30 Chinese provinces from 2012 to 2021. By employing a coupling coordination model and dynamic fuzzy-set qualitative comparative analysis (fsQCA), this study systematically reveals the interaction mechanisms and optimization pathways between the two systems.

**Results:**

The key findings are as follows: (1) Temporal Trends: The degree of coupling coordination has undergone a phased transition from “on the verge of disorder” to “primary coordination.” However, the overall growth remains limited, indicating an urgent need to shift from a “scale expansion” model to a “quality-driven” approach. (2) Spatial Patterns: A distinct regional disparity is observed, characterized by an “eastern leading, central catching up, and western lagging behind” pattern. Notably, 80% of provinces in central and western China remain constrained by digital economy-induced maladaptation. (3) Spatial Correlation: The coupling coordination degree exhibits significant positive spatial clustering characteristics. Provinces such as Anhui and Hubei in central China have achieved leapfrog development by leveraging technological spillovers. (4) Driving Mechanisms: The fsQCA results identify three distinct high-coordination configurations: the “digital infrastructure-driven” model in eastern China, the “government–human capital dual-driven” model in central China, and the “government–institutional environment synergy-driven” model in western China.

**Discussion:**

These findings underscore the necessity for region-specific development strategies that align with local resource endowments and contextual factors. By adopting differentiated policy pathways, provinces can effectively promote the coupling and coordinated development of essential public health services and the digital economy, ultimately fostering a high-quality and sustainable integration of the two systems.

## 1 Introduction

The report of the 20th National Congress of the Communist Party of China explicitly states that “high-quality development is the primary task of building a modern socialist country in all respects.” The equalization and accessibility of essential public health services serve as critical benchmarks for evaluating the quality of development (1). However, China continues to face significant challenges in delivering these services, including disparities in resource distribution, inefficiencies in service provision, and regional technological imbalances (2). In response, both the 14th Five-Year Plan for Digital Economy Development and the Implementation Plan for Promoting Common Prosperity through the Digital Economy emphasize the necessity of leveraging digital technologies to restructure the provision of essential public health services. This strategic approach integrates “technological empowerment” and “institutional innovation” as dual drivers to mitigate the challenges posed by unbalanced development (3). The digital economy and essential public health services constitute two interdependent systems, engaging in a dynamic and mutually reinforcing process of coordinated development rather than a unidirectional causality. On one hand, the digital economy enhances public health service systems by leveraging technological innovation and data-driven empowerment to optimize resource allocation and enable precise healthcare delivery (4). On the other hand, the continuous improvement of essential public health services expands application scenarios for the digital economy, generating new market demands and propelling its evolution toward higher stages of development. Therefore, it is imperative to assess the degree of coordination between the digital economy and essential public health services across different regions in China and to examine the underlying influencing factors. Such an investigation is of great significance for fostering their synergistic development.

Existing research on this topic primarily falls into two categories. The first stream of research explores how the digital economy facilitates the advancement of essential public health services. Empirical studies indicate that the digital economy reduces the cost of public health service provision through technological innovation, human resource optimization, and enhanced data management capabilities (5, 6), while significantly improving service efficiency (7), thereby reinforcing the development of essential public health services (8, 9). With the maturation of policies and the deepening of research, scholars have increasingly acknowledged the pivotal role of the digital economy in optimizing healthcare systems, enhancing resource allocation efficiency, and improving service accessibility (10). Initial discussions within the public health domain have also emerged. For instance, Li et al. (8) employed a mediation effect model and demonstrated that the digital economy not only directly enhances urban public health outcomes but also generates significant spatial spillover effects. Zhao et al. (11) analyzed the impact pathways of digital economic development on public health services, focusing on social media usage and urban–rural disparities in health insurance coverage. Guan et al. (11, 12) utilized a bidirectional fixed-effects model to examine the impact of digital economic development on the efficiency of essential public health services. Bao et al. (13) underscored the crucial role of digital media in disseminating visualized health information, coordinating healthcare resources through mobile health applications, facilitating public health campaigns via social media, and supporting population health management and disease surveillance through digital tools. Furthermore, Lyu et al. (7) highlighted that the digital economy enhances public health service efficiency by improving governmental performance and regulatory quality. The second stream of research examines how the development of essential public health services contributes to the expansion of the digital economy. Existing studies suggest that the digital transformation of public health services reciprocally drives digital economic growth through a multidimensional “demand-driven—technology response” co-evolution mechanism (14). Within this framework, the increasing demand for digitalized public health services stimulates developments in the data element market, information infrastructure construction, and medical technology innovation, thereby accelerating the formation and optimization of the digital economic system. On one hand, the digitalization of public health services fosters the expansion of the data element market and facilitates the restructuring of the digital economy’s value chain through the standardization and openness of health data. Large-scale health data generated within public health services—including electronic health records, chronic disease management data, vaccination records, and genomic sequencing data—can be widely applied in AI-driven medical diagnostics, precision medicine, and personalized health management, provided that data desensitization and privacy protection measures are effectively implemented (15). On the other hand, the digital transformation of essential public health services directly drives the advancement of next-generation infrastructure technologies, such as 5G, the Internet of Things (IoT), and cloud computing, thereby providing foundational technical support for the digital economy (16). Additionally, the establishment of a nationally unified health data standard further accelerates the standardization process for electronic medical records, medical imaging, and genomic data, laying the groundwork for the digital health industry’s infrastructure (17). Moreover, the intelligent upgrading of essential public health services has catalyzed the emergence of new business models within the digital economy, particularly in the Internet + Healthcare sector (18). A prominent example is the rise of Internet hospitals, which integrate online consultations, electronic prescriptions, pharmaceutical distribution, and medical insurance payments, thereby realizing end-to-end digitalization of healthcare services (19). This model not only alleviates the structural mismatch between offline healthcare supply and demand but also stimulates the development of related industries, including pharmaceutical e-commerce, health insurance, and intelligent health management.

In recent years, as scholarly research on the digital economy and essential public health services has advanced, both domestic and international studies have primarily concentrated on three key areas: the development of evaluation indicators, the assessment of current conditions, and the exploration of underlying mechanisms (20, 21). Methodologically, prior research has predominantly employed panel regression models, efficiency models, coupling coordination models, and Gini coefficients to examine the interactions between essential public health services and various socioeconomic and demographic factors (21–25). However, limited attention has been given to investigating the factors influencing the coordinated development of essential public health services and the digital economy from a configurational perspective. Existing literature suggests that, with appropriate governmental support, the expansion of the digital economy can significantly enhance the development of public health services. Conversely, high-quality public health services serve as a crucial foundation for shaping the trajectory of digital economic growth (26). However, in some regions, short-term economic interests may be prioritized, leading to increased investment in digital economic development at the expense of long-term public health benefits (12). This imbalance not only results in time-lag effects but also disrupts the positive interplay between essential public health services and the digital economy. Therefore, in the digital era, achieving high-quality public health service provision requires a balanced approach that integrates both domains. Leveraging digital technologies to reduce regional disparities and promote the equitable distribution of essential public health services is essential.

While existing studies recognize that the digital economy can strengthen the healthcare system by reducing service costs, enhancing medical efficiency, and optimizing health governance capabilities (22, 23), several critical limitations remain: (1) A predominant focus on the unidirectional impact of the digital economy on public health services, while neglecting their bidirectional interaction, particularly how evolving public health demands drive iterative advancements in digital technologies. (2) The absence of systematic assessments of the coordinated development level between these two domains, especially regarding spatial heterogeneity and temporal evolution dynamics. (3) An overreliance on regression analysis, which is insufficient to capture the intricate pathways and conditional configurations influencing coupling coordination. Consequently, policy recommendations often default to broad-stroke strategies, such as generalized investment increases, without considering regional adaptability.

To address these research gaps, this study adopts the WSR (Wuli-Shili-Renli) system methodology to construct a comprehensive three-dimensional analytical framework encompassing the physical layer (digital infrastructure), the institutional layer (policy coordination), and the human layer (demand response). By employing a bidirectional coupling perspective, this study seeks to elucidate the co-evolutionary mechanisms governing the digital economy and public health services. Methodologically, it integrates an enhanced coupling coordination model with dynamic fuzzy-set qualitative comparative analysis (fsQCA) to achieve the following key contributions: (1) Theoretical Contribution: Unveiling the closed-loop interaction pattern of “technological supply–institutional incentives–demand-driven forces,” thereby addressing the limitations of unidirectional causal explanations and deepening the understanding of their interactive mechanisms. (2) Methodological Innovation: Identifying spatial–temporal heterogeneity and configurational pathways to overcome the limitations of “averaged” conclusions in conventional research, thus providing empirical support for regionally differentiated policymaking. (3) Policy Implications: Proposing a region-specific intervention strategy of “technological deepening in the East, resource integration in the Central region, and compensatory innovation in the West.” This approach tailors regulatory measures to regional characteristics, transitioning cross-system governance from a “one-size-fits-all” model to a more precise, targeted framework.

This study not only clarifies how the digital economy can facilitate the equitable provision of essential public health services but also offers policymakers a scientific basis for optimizing resource allocation, reducing regional disparities, and fostering the synergistic development of the digital economy and public health services. Ultimately, it aims to support the high-quality implementation of the “Healthy China” strategy.

## 2 Theoretical mechanism

### 2.1 Coupled coordination mechanism

The theory of coupling and coordination encompasses two key concepts: “coupling” and “coordination.” Coupling refers to the mutual influence and interdependence between systems, wherein two or more systems interact through dynamic correlation, characterized by continuous bidirectional influence rather than unidirectional causality. Coordination, in contrast, emphasizes the constructive interaction and symbiotic development between systems through the transmission and transformation of matter, information, and energy (27). As a system transitions from a disordered to an ordered state, the concepts of coupling and coordination together reflect the interactions among constituent elements or systems, as well as their developmental dynamics (28). Utilizing the theory of coupling and coordination, the interplay between the digital economy and essential public health services can be understood as an interactive process in which the two systems support and influence each other. A close coupling and coordination relationship exists between the digital economy and essential public health services, wherein their interactions jointly promote the efficient, intelligent, and sustainable development of public health. The digital economy provides technical support for the equalization of essential public health services, while an efficient essential public health service system offers health data and market demand that drive continuous innovation within the digital economy, thereby establishing a virtuous cycle of interdependence and mutual enhancement.

#### 2.1.1 Digital economy creates conditions for achieving the goal of equalization of essential public health services

The digital economy, underpinned by digital governance and supported by digital infrastructure, facilitates the deep integration of industrial digitization and digital industrialization through the acceleration of digital technology innovation and application. It encompasses five key dimensions: digital infrastructure, digital industrialization, industrial digitization, digital innovation, and digital governance (29). Accordingly, the promotion of the digital economy’s impact on essential public health services is primarily evident in the following five aspects: (1) “Digital Infrastructure”: The construction of digital infrastructure effectively eliminates the spatial and temporal limitations of traditional services, providing a rapid and convenient channel for the cross-regional flow of capital, technology, and talent, thereby optimizing the allocation of healthcare resources (30). Furthermore, with the continuous enhancement of digital infrastructure, the speed of information dissemination can be significantly improved, mitigating the phenomenon of information asymmetry in the market (31). This not only facilitates the aggregation of demand and supply information for essential public health services but also enables dynamic matching and precise alignment of supply and demand, thereby accelerating the flow of resource elements and reducing transaction costs associated with information asymmetry. (2) “Digital Industrialization”: An increase in the level of digital industrialization enhances the capacity to diversify the supply of digital products, thereby promoting the digital development and application of these products within the healthcare sector and ultimately improving the supply capacity of essential public health services (32). (3) “Industrial Digitization”: The digital transformation of traditional industries enhances the accessibility and convenience of essential public health services. Moreover, the integration of the digital economy with traditional industries has injected new impetus into the development of essential public health services, broadening financing channels and optimizing the financing structure for these services (33). (4) “Digital Innovation”: Digital innovation fosters the development of essential public health services by accelerating the integration of digital technology with medical technology, enhancing equipment and facility upgrades, and improving the overall quality and efficiency of these services (34). (5) “Digital Governance”: Digital governance establishes a robust institutional guarantee and development environment for the high-quality advancement of essential public health services by enhancing government transparency, increasing administrative efficiency, and strengthening regulatory effectiveness (35). Additionally, the inclusive nature of the digital economy allows remote areas and disadvantaged groups greater access to medical resources and service opportunities, thereby improving the equity and accessibility of essential public health services. With the aid of telemedicine systems and mobile health applications, residents can transcend geographic constraints and enjoy more equitable essential public health services, ultimately improving service coverage and enhancing social welfare balance (36).

#### 2.1.2 Essential public health services support the high-quality development of the digital economy

The developmental goals of essential public health services primarily focus on enhancing the health of the entire population, promoting health equity, optimizing the public health system, advancing informatization and intelligence, and responding to emerging health challenges. The ultimate aim is to comprehensively improve population health and ensure the efficient and sustainable development of public health services, considering both economic and social benefits (37, 38). Thus, the promotion of essential public services to the digital economy is evident in the following two aspects: (1) “Economic Benefits”: The high-quality development of the digital economy is inextricably linked to the support provided by health data and the promotion of smart medical services. Continuous enhancements in essential public health services bolster the efficiency of health data collection and utilization, thereby promoting the growth of the digital health industry, including health big data, mobile health applications, telemedicine, and wearable devices. This linkage not only improves the level of essential public health services but also fosters the digital transformation of related industries, which in turn stimulates regional economic growth and establishes a robust foundation for the digital economy’s advancement (32). Additionally, the equalization of essential public health services enhances the efficiency of grassroots medical services, alleviating pressure on medical resources in urban centers, optimizing the overall allocation of medical resources, and indirectly expanding market demand for healthcare-related digital services (39). (2) “Social Benefits”: Firstly, the digital transformation of essential public health services relies on the support of digital technologies such as telemedicine and health monitoring platforms, which impose higher demands on the digital economy’s infrastructure, consequently driving innovation and application of these technologies (40). Secondly, high-quality public health services improve population health and reduce the risk of disease transmission, thereby ensuring stable economic operation and creating favorable conditions for the digital economy to attract talent and resources (41). Finally, the ongoing optimization of essential public health services enhances the health environment, further accelerating the sustainable development of the digital economy. Digital health products derived from changes in essential public health services, including wearable devices, mobile health management platforms, and electronic health records, have not only improved the quality of essential public health services but also facilitated the penetration and expansion of the digital economy within the health sector (42). The digital economy achieves high-quality development through coordinated efforts across various fields such as drug research and development, personalized medical services, and health insurance. The development of essential public health provides rich application scenarios for the digital economy while promoting balanced digital technology advancement across regions, thus creating conducive conditions for narrowing the digital divide between urban and rural areas.

**Figure 1:**
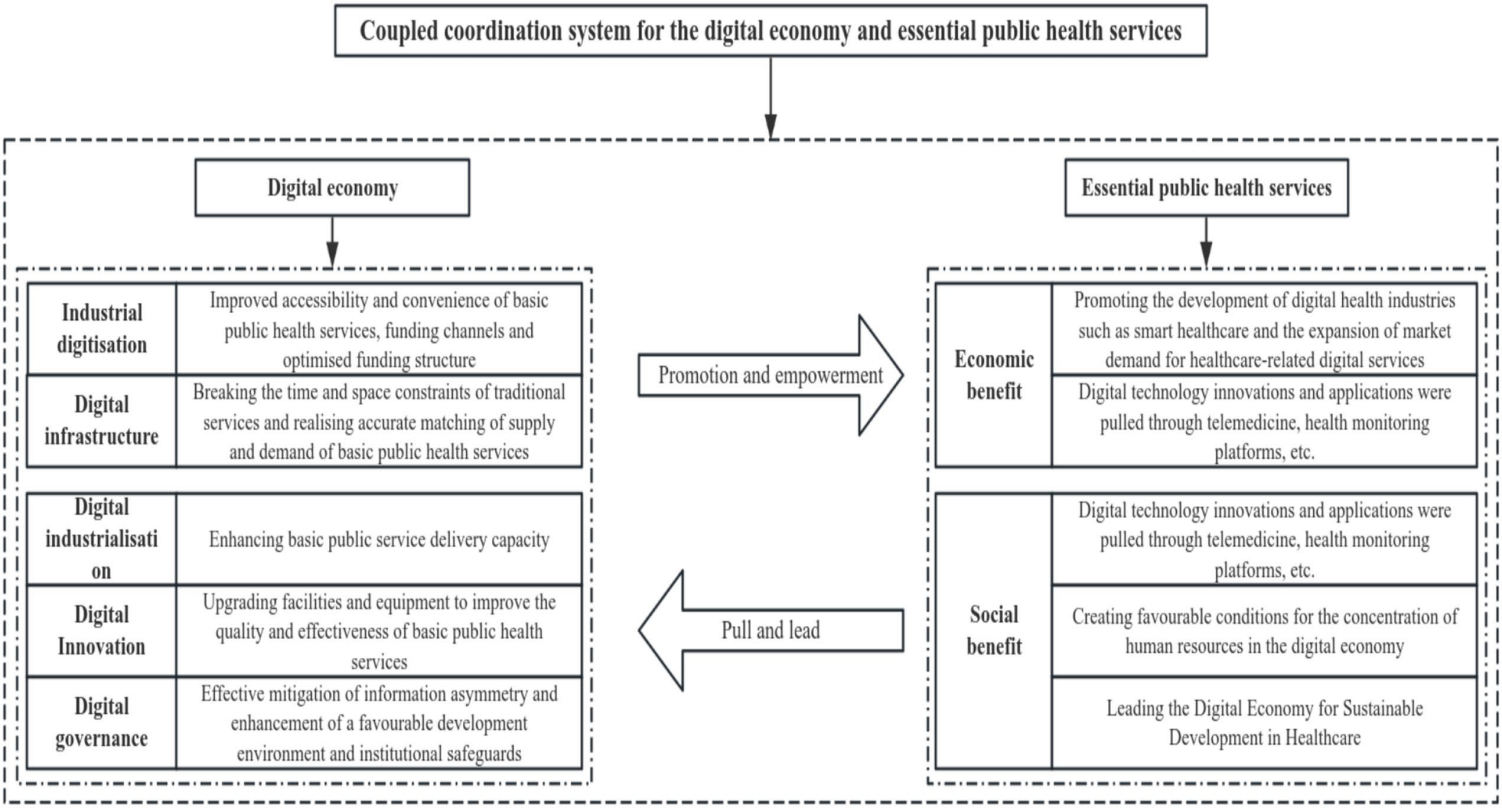
Theoretical model.

The coupling mechanism between the digital economy and essential public health services is characterized by mutual promotion and interdependence. Digital technology enhances the universality and equity of essential public health services, while the health data and market demand generated by the essential public health service system provide significant impetus for the innovative development of the digital economy. This synergistic development not only aids in improving residents’ health status and overall social wellbeing but also offers new perspectives for constructing an essential public health service system in the digital era, ultimately realizing the goal of coordinated development between China’s essential public health service and digital economy. Consequently, this paper clarifies the coupling relationship between the digital economy system and the essential public health service system, constructing a coupling coordination model for both systems (Figure 1).

## 3 Materials and methods

### 3.1 Research methodology

#### 3.1.1 Coupling evaluation model

In the coupling evaluation model, although the traditional coupling degree analysis can reflect the interactions generated between the two systems, it is difficult to discern the magnitude of synergistic utility. Therefore, this paper introduces the coupling coordination index, and regards public health service and digital economy as two different systems in order to construct the coupling coordination model of essential public health service and digital economy (43), as shown in Equations 1–3:


(1) $$C=\frac{2\sqrt{U_{1}U_{2}}}{\left(U_{1}+U_{2}\right)}$$

(2) $$D=\sqrt{C\times T}$$

(3) $$T=\alpha U_{1}+\beta U_{2}$$

Where C is the degree of coupling, D is the degree of coupling coordination, T is the comprehensive evaluation index of the two systems, U_1_ and U_2_ are the evaluation values of essential public health services and digital economy, respectively; α and β are the coefficients to be determined, and they satisfy α = β = 0.5 (44), which indicates that the essential public health services are equally important as the digital economy. In order to more intuitively reflect the coupled and coordinated development of essential public health services and digital economy in each region, this paper refers to the relevant research results of Li (39) to divide the degree of coordinated development of the two subsystems, as shown in Table 1.

**Table 1:** Coupling coordination evaluation criteria.

Coordination type	Coupling coordination degree D value range	Coupling coordination degree
Disordered decline	(0.0–0.1)	Extremely disordered
	[0.1–0.2)	Serious disorder
	[0.2–0.3)	Moderate disorder
	[0.3–0.4)	Mild disorder
Transitional harmony	[0.4–0.5)	Nearly disorder
	[0.5–0.6)	Barely coordinated
Coordinated development	[0.6–0.7)	Primary coordination
	[0.7–0.8)	Intermediate coordination
	[0.8–0.9)	Good coordination
	[0.9–1.0)	High-quality coordination

### 3.2 WSR methodology

The WSR methodology (Wuli-Shili-Renli methodology) is a theoretical framework rooted in Chinese philosophy and widely applied in management and systems science. It conceptualizes the analysis of systems through three core dimensions: “things” (Wuli), “matters” (Shili), and “people” (Renli), addressing these factors from physical, practical, and humanistic perspectives. The WSR methodology emphasizes the holistic interconnections, mutual constraints, and interactions among the physical (W), social (S), and human (R) dimensions. It aims to resolve complex problems by focusing on the physical attributes of the object, formulating optimal strategies to address challenges, and employing a systematic management and decision-making framework informed by human cognition (45). Given that the coupling and coordination of essential public health services and the digital economy are characterized by systemic complexity, WSR offers an appropriate theoretical framework by providing insights from the physical, practical, and human dimensions (Table 2).

**Table 2:** A collection of indicators for evaluating inter-provincial the essential public health services and the digital economy in China.

Target system	Tier 1 indicators	Tier 2 indicators	Orientations
Essential public health services	Medical resource input	Grassroots medical and health institutions per thousand people	+
		Number of beds in health institutions per thousand people	+
		Number of professional (assistant) physicians per thousand people	+
	Medical services output	Per capita total health expenses	+
		Resident hospitalization rate	+
		Maternal mortality rate	−
		Newborn visit rate	+
	Health prevention services	Incidence rate of Class A and B infectious diseases	−
		Premarital medical examination rate	+
		Women’s disease examination rate	+
		Number of public health education activities per thousand people	+
	Health protection services	Medical insurance coverage rate	+
		Number of health examinations (person times per thousand people)	+
		The maternity insurance coverage rate	+
Digital economy	Digital infrastructure	Broadband access ports for Internet use per 100 population	+
		Mobile phone penetration rate	+
		IPv4 addresses per 100 population	+
		Domain names per 100 people	+
		Web pages per 100 inhabitants	+
		Mobile phone base stations per 100 population	+
		Fiber optic cable route density	+
	Digital industrialization	Total telecommunication services per capita	+
		Percentage of employees in the information transmission, software industry	+
		Software product revenue per capita	+
		Software product revenue per capita operating income of electronic information manufacturing enterprises	+
		Number of manufacturing enterprises in the electronic information industry	+
		Number of enterprises in the software and information technology services industry	+
		Percentage of digital TV subscribers	+
	Industrial digitization	Percentage of employees in computer services and software	+
		Percentage of rural broadband access users	+
		E-commerce transactions per capita	+
		Computers per 100 population	+
		Websites per 100 enterprises	+
		Digital Inclusive Finance Index	+
	Digital Innovation	R&D expenditure on software and information technology services	+
		Software and information technology services R&D staff	+
		R&D personnel in the electronics and communications equipment manufacturing industry	+
		R&D expenditure on electronics and communications equipment manufacturing	+
		Internal expenditures	+
	Digital Governance	Government online government service capacity index	+

#### 3.2.1 Physical dimension

The physical (Wuli) dimension concerns the natural, technical, or structural aspects of the system and how it operates. It focuses on how a system is structured, functions, and is technically realized (46). In this study, the physical dimension refers to the exogenous, long-term stable, and objective institutional environment that influences the coupling and coordination between essential public health services and the digital economy. A robust institutional environment is the foundation for facilitating technological innovation, cooperative initiatives, and regional resource allocation, thus enhancing regional essential public health services (47). Consequently, this study considers the institutional environment a key physical condition affecting the coupled and coordinated development of essential public health services and the digital economy.

#### 3.2.2 Matter-of-fact dimension

The matter-of-fact (Shili) dimension involves the logic, processes, and operational rules of the system, addressing how problems are handled within the system’s internal regulations (46). It reflects decision-makers’ strategies in response to external environments and internal conditions. In the context of coupling and coordinating essential public health services and the digital economy, the matter-of-fact dimension includes intervention methods, implementation strategies, and management processes. Government support is a critical factor in stimulating and regulating the coupled development of these two systems, often providing systems and funding to facilitate their coordination (48). This study therefore identifies government support as a key matter-of-fact condition in influencing the coupling and coordination of essential public health services and the digital economy.

#### 3.2.3 Humanistic dimension

The humanistic (Renli) dimension focuses on the human elements within the system, such as interaction, culture, values, and the emotions that affect system operations. It emphasizes the relationships among actors and their role in organizing effective practices (46). In the process of coupling essential public health services and the digital economy, humanistic factors involve the willingness, ability, and motivation of various stakeholders to engage in collaborative activities (46). Human capital plays a pivotal role in creating both economic and social value (49). From the perspective of collaborative innovation theory, higher levels of human capital encourage cooperation among actors, resulting in a synergistic effect that generates co-created value unattainable by individuals acting alone (50). Therefore, this paper views human capital as a critical humanistic factor in the coupling and coordinated development of essential public health services and the digital economy.

WSR methodology argues that the physical, factual, and humanistic dimensions are interrelated and interact dynamically rather than existing as isolated elements (51). Addressing systemic problems requires not only focusing on objective realities (W) but also considering how interventions (S) are shaped by human cognition, as human factors ultimately influence the effective organization of ‘things’ and ‘matters’. Thus, it is essential to account for the interrelations and interactions among all actors and their change processes (R) to organize the most effective practices that maximize system efficiency (52, 53).

**Figure 2:**
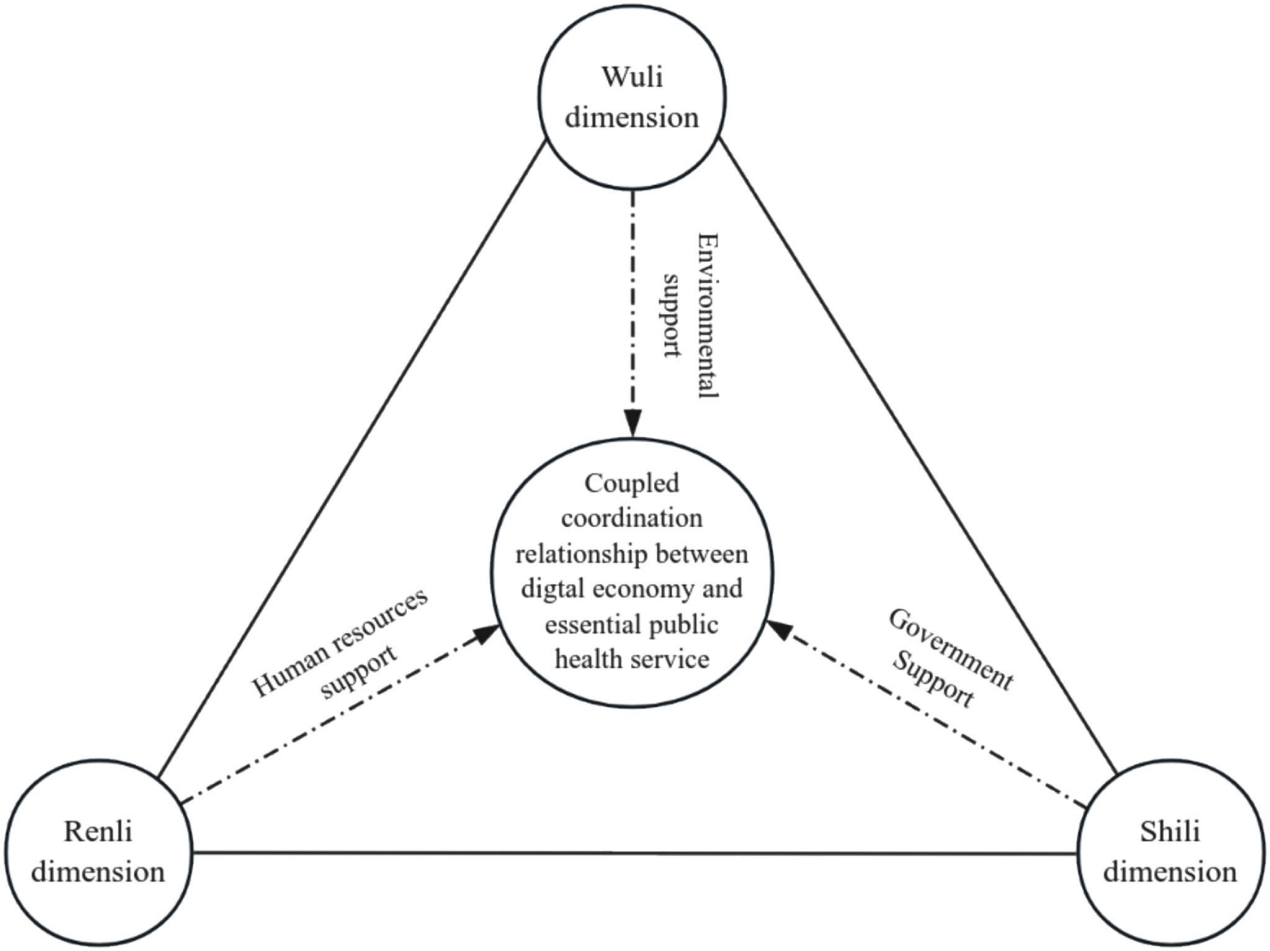
The configuration model.

From a WSR perspective, the institutional environment determines the resource dependency path of system actors (46), representing an objective condition (W). Government support, essential for the coupled development of the two systems, is influenced by institutional constraints and the level of human capital (54). Human capital reflects the extent to which the institutional environment and government support can be leveraged to promote coupling and coordinated development, necessitating consideration of how system actors’ capabilities (R) can enhance the triadic interaction of “things,” “matters,” and “people.” This interaction aims to achieve the optimal state of coupled development between the two systems (55). Moreover, WSR methodology does not operate in isolation but rather synergistically across the three dimensions—physical, factual, and human. Therefore, this paper constructs The configuration model shown in Figure 2, guided by WSR methodology, to explore how the antecedent conditions across these three dimensions interact and align to influence the coupled and coordinated development of essential public health services and the digital economy.

#### 3.2.4 Fuzzy-set qualitative comparative analysis

Fuzzy-set Qualitative Comparative Analysis (fsQCA) is a research method that integrates both qualitative and quantitative approaches, and is commonly applied in social sciences, management, and political science to analyze complex causal relationships involving multiple factors or conditions. fsQCA leverages the strengths of both case study and variable-based approaches to uncover aggregate relationships between factor configurations and outcomes through set-theoretic analysis (56). This method is particularly suited for addressing grouping problems in complex systems, making it an ideal tool for analyzing the development paths of the coupled and coordinated relationship between the essential public health services and the digital economy. Firstly, the coupling between the essential public health services and the digital economy is influenced by multiple conditions. fsQCA explores the relationships between these conditions and outcomes through a configurational approach. Secondly, while traditional empirical methods, such as regression analysis, typically examine the net effect of individual variables, fsQCA is capable of identifying multiple equivalent configurations that lead to similar outcomes. Finally, fsQCA is well-suited to addressing grouping issues in complex systems, making it an ideal tool for examining the development trajectories of the coupling between the essential public health services and the digital economy.

### 3.3 Variable selection

#### 3.3.1 Constructing evaluation indicators for the essential public health services and the digital economy

Currently, there is no standardized approach to measure the level of digital economy development. Compared to value-added accounting methods and word frequency analysis of policy texts, the approach of constructing an indicator system provides a more comprehensive view of various aspects of digital economy development. In contrast to the compilation of satellite accounts, indicator-based measurement is more concise, intuitive, and facilitates easier comparison and analysis (57). Consequently, this paper draws on existing research to develop an evaluation index system for digital economy development (58–60). This system includes 26 key indicators across five dimensions: digital infrastructure, digital industrialization, industrial digitization, digital innovation, and digital governance. Among them, digital infrastructure is the cornerstone of the development of the digital economy, as measured by indicators such as the number of broadband access ports for Internet use per 100 people and the mobile phone penetration rate. Digital industrialization reflects the level of development of industries relying on digital technology and data elements, and is measured through indicators such as the total telecommunication services per capita and percentage of employees in the information transmission, software industry. Industrial digitization measures the degree of integration between the digital economy and traditional industries, as measured by indicators such as the percentage of employees in computer services and software, and the percentage of rural broadband access users. Digital innovation reflects the investment in digitally related R&D personnel and R&D funding, measured through indicators such as R&D expenditure on software and information technology services and software and information technology services R&D staff. Digital governance is the application of digital technology in government governance, reflecting the effectiveness of the digital government’s government services, as measured by the index of the government online government service capacity index. For essential public health services, drawing on relevant research, this study selects 14 core indicators across four dimensions: medical resource input, medical service output, health prevention services, and health protection services (61–63). Among them, the medical resource input and medical service output reflect the economic benefits of essential public health services, as measured by such indicators as the grassroots medical and health institutions per thousand people, the number of beds in health institutions per thousand people, the per capita total health expenses and the maternal mortality rate. The dimensions of health prevention and health protection services reflect the social benefits of essential public health services, as measured by such indicators as the incidence rate of class A and B infectious diseases, the rate of premarital medical examination rate, the medical insurance coverage rate and the number of health examinations (61). Accordingly, this study constructs an evaluation index system for the coupling and coordinated development of the digital economy and essential public health services, as presented in Table 1. In multi-indicator comprehensive evaluations, discrepancies in the dimensional scales and magnitudes of the original indicators necessitate a dimensionless transformation to eliminate unit inconsistencies and enhance data comparability. To objectively determine the weight of each indicator, the entropy method is employed to calculate the development indices for both essential public health services and the digital economy. The specific steps are as follows:

(1) dimensionless transformation:

$$\mathrm{Positive indicators}:X_{ij}^{*}=\frac{x_{ij}-\min\left(x_{ij}\right)}{\max\left(x_{ij}\right)-\min\left(x_{ij}\right)}$$

$$\mathrm{Negative indicators}:X_{ij}^{*}=\frac{\max\left(X_{ij}\right)-X_{ij}}{\max\left(X_{ij}\right)-\min\left(X_{ij}\right)}$$

Where $X_{ij}^{*}$ is the normalized data and $X_{ij}$ is the original value of the jth sample on index i. i=1,2,3,…,n , j=1,2,3,…,n . Because the standardized data may have a value of 0, to reduce its impact on the calculation, all standardized data are shifted by 0.0001.

(2) calculation of indicator proportion: $P_{ij}=\frac{x_{ij}^{*}}{\overset{n}{\underset{i=1}{\sum }}x_{ij}^{*}}$
(3) calculate the information entropy. The formula is as follows: $e_{j}=-k\overset{n}{\underset{i=1}{\sum }}\left(P_{ij}\times \ln P_{ij}\right),e_{j}\geq 0$
(4) calculation of the utility of the indicator: $d_{j}=1-e_{j}$
(5) calculation of index weights: $W_{j}=\frac{d_{j}}{\overset{m}{\underset{j=1}{\sum }}d_{j}}$
(6) calculate the composite index score: $\mathrm{SCOR}E_{i}=\overset{m}{\underset{i=1}{\sum }}W_{j}\times X_{ij}^{*}$

#### 3.3.2 Drivers of coupled coordination between the digital economy and essential public health services

##### 3.3.2.1 Result variables

Exploring the relationship between the coupled coordination of the digital economy and essential public health services holds significant practical value for promoting the synergistic development of both sectors. Therefore, this study uses the degree of coupled coordination between the digital economy and essential public health services as the outcome variable, reflecting the level of coordination between the two systems.

##### 3.3.2.2 Conditional variables

Based on the WSR framework, the influencing factors for the development of the digital economy and essential public health services are analyzed across three dimensions:

Institutional Environment (W): A well-adapted institutional environment is a key driver of China’s economic development, fostering technological advancement and facilitating institutional reforms (47). The externalities produced by a robust institutional environment can further accelerate digital technology development (56). Drawing on Wang Xiaolu’s study (64), this paper employs the secondary index of the marketization index to assess the adaptability of the institutional environment.

Government Support (S): The government plays a pivotal role in advancing the coordinated development of the digital economy and essential public health services. By leveraging fiscal expenditure to mitigate external uncertainties, the government fosters digital technology innovation, which, in turn, creates conducive conditions for the coordinated development of both systems (65). Consequently, this paper uses fiscal expenditure on science and technology (log-transformed) as an indicator of government support.

Human Capital (R): According to innovation theory, regions with richer human capital exhibit higher degrees of knowledge heterogeneity and greater capacities to enhance digital economy technologies (63). Human capital, as a critical resource, supports technological advancement and strengthens the level of coordination between the digital economy and essential public health services. As such, this paper uses the ratio of students enrolled in higher education to the total population at year-end as a measure of human capital.

### 3.4 Data source

During the period 2012–2021, the Chinese government has implemented a series of policies to improve the efficiency of public health services, equalize medical resources and reduce the gap between urban and rural areas (60). Since 2012, a more comprehensive statistical system has been established and data on the digital economy (e.g., Internet penetration rate, e-commerce turnover, etc.) and public health services have been more completely documented during this time period. These policies provide an important support for promoting the efficient and intelligent development of the digital economy and essential public health services. Therefore, 30 provinces, autonomous regions, and municipalities directly under the central government of China (excluding Tibet, Hong Kong, Macao Special Administrative Region, and Taiwan) are selected for this paper for the period of 2012–2021, and the indicators selected for the study are derived from the China Statistical Yearbook, the China Health Statistical Yearbook, and the statistical yearbook data of each province. The China Health Statistics Yearbook provides data on China’s public health services, and the China Statistics Yearbook provides data on China’s digital economy and social development. In addition, this paper divides China into eastern, central and western regions based on the division method of the China Statistical Yearbook.

## 4 Analysis of results

### 4.1 Analysis of the coupling and coordination degree between the digital economy and essential public health services

**Figure 3:**
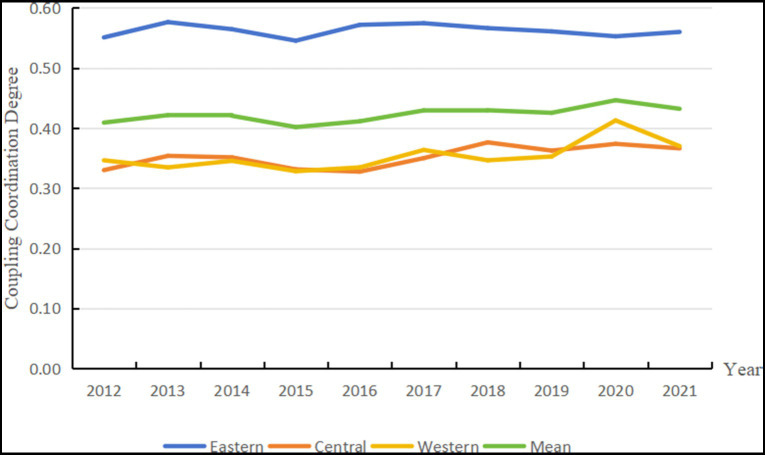
Trends in the degree of coordination of the coupling of the two systems in China’s provinces and cities.

At the national level, Figure 3 illustrates that the average coupling and coordination degree between the digital economy and essential public health services across China’s provinces and cities exhibited a slight upward trend from 2012 to 2021. This value increased from 0.41 in 2012 to 0.43 in 2021, representing an average annual growth rate of 0.55%. Overall, the coupling and coordination degree remains within the range between “on the verge of dysfunction” and “barely coordinated,” suggesting that the current interaction between China’s digital economy and essential public health services requires further strengthening. The coupled and coordinated development of these two systems remains in need of significant improvement.

At the regional level, the average annual increase in the coupling and coordination degree for the eastern, central, and western regions during the same period was 0.16, 1.05, and 0.66%, respectively. The eastern region consistently maintained a level of “barely coordinated,” which reflects a transitional and reconciliation phase. In contrast, both the central and western regions, although showing improvements compared to their 2012 levels, remained below the national average and are classified as being in the “dysfunctional” and “recessionary” stages. A possible explanation for these regional disparities is that the eastern region benefits from resource advantages over the central and western regions, with more comprehensive digital infrastructure and public health service systems. Consequently, the levels of digital economy development and the coordinated progress of essential public health services are comparatively lower in the central and western regions.

At the city level, as shown in Table 3, the degree of coupling and coordination between the digital economy and essential public health services varied significantly across Chinese provinces from 2012 to 2021. Most cities experienced a decline in coupling coordination between 2019 and 2021, with increasing spatial differentiation becoming more apparent. The vast majority of cities fell into the category of “over-adjustment.” Notably, only six regions—Beijing, Shanghai, and Jiangsu Province among them—achieved the coordinated development stage during this period, reaching the levels of “primary coordination” or “good coordination.” Relative to cities with lower coupling levels, these cities have abundant human, material and policy resources that can deeply promote the integration and development of the digital economy and essential public health services. For example, in the context of government guidance, higher levels of economic development and improved infrastructure provide effective support for promoting the application of digital technologies, such as the Internet and 5G, in the field of public health services, thus facilitating the deep integration of the digital economy and essential public health services.

**Table 3:** Degree of coupling and coordination between essential public health services and digital economy in China’s provincial areas, 2012–2021.

Region	Province	2012	2013	2014	2015	2016	2017	2018	2019	2020	2021
Eastern	Beijing	0.84	0.92	0.92	0.83	0.84	0.92	0.84	0.93	0.84	0.83
	Tianjing	0.23	0.22	0.21	0.21	0.22	0.23	0.24	0.24	0.24	0.25
	Hebei	0.48	0.49	0.47	0.32	0.47	0.51	0.50	0.48	0.49	0.50
	Liaoning	0.51	0.51	0.50	0.47	0.50	0.50	0.47	0.44	0.44	0.43
	Shanghai	0.61	0.75	0.74	0.73	0.75	0.63	0.64	0.64	0.65	0.66
	Jiangsu	0.61	0.63	0.61	0.68	0.71	0.66	0.72	0.66	0.66	0.68
	Zhejiang	0.68	0.68	0.67	0.69	0.70	0.71	0.70	0.71	0.70	0.71
	Fujian	0.54	0.54	0.53	0.52	0.53	0.54	0.53	0.53	0.53	0.54
	Shandong	0.55	0.58	0.56	0.55	0.58	0.58	0.56	0.54	0.55	0.58
	Guangdong	0.80	0.81	0.79	0.79	0.78	0.81	0.80	0.78	0.76	0.75
	Hainan	0.21	0.21	0.21	0.21	0.21	0.23	0.23	0.22	0.22	0.23
Central	Shanxi	0.45	0.52	0.43	0.39	0.19	0.19	0.41	0.40	0.41	0.41
	Jilin	0.21	0.20	0.20	0.18	0.20	0.21	0.20	0.17	0.15	0.14
	Heilongjiang	0.40	0.41	0.43	0.39	0.42	0.20	0.18	0.17	0.16	0.12
	Anhui	0.13	0.14	0.15	0.15	0.17	0.40	0.41	0.41	0.43	0.43
	Jiangxi	0.27	0.30	0.34	0.32	0.34	0.39	0.39	0.36	0.38	0.39
	Henan	0.27	0.35	0.35	0.36	0.41	0.46	0.47	0.45	0.48	0.49
	Hubei	0.49	0.51	0.51	0.50	0.51	0.52	0.52	0.53	0.53	0.52
	Hunan	0.42	0.40	0.40	0.36	0.38	0.43	0.43	0.41	0.45	0.43
Western	Neimenggu	0.22	0.22	0.21	0.19	0.20	0.21	0.21	0.19	0.41	0.39
	Guangxi	0.37	0.36	0.34	0.32	0.32	0.36	0.40	0.36	0.48	0.38
	Chongqing	0.42	0.44	0.44	0.44	0.45	0.48	0.47	0.47	0.47	0.47
	Sichuan	0.50	0.55	0.55	0.58	0.59	0.61	0.60	0.60	0.62	0.61
	Guizhou	0.23	0.23	0.23	0.23	0.27	0.41	0.38	0.38	0.38	0.23
	Yunnan	0.32	0.37	0.36	0.36	0.37	0.44	0.38	0.38	0.46	0.27
	Shanxi	0.48	0.47	0.47	0.47	0.49	0.50	0.51	0.49	0.50	0.49
	Gansu	0.12	0.14	0.13	0.14	0.13	0.18	0.17	0.18	0.18	0.19
	Qinghai	0.43	0.18	0.39	0.18	0.18	0.21	0.21	0.19	0.41	0.38
	Ningxia	0.19	0.19	0.19	0.19	0.19	0.22	0.21	0.21	0.21	0.21
	Xinjiang	0.53	0.53	0.49	0.51	0.49	0.38	0.27	0.43	0.42	0.45

In terms of development trends, Anhui Province demonstrated a significantly higher improvement in the coupling coordination degree than other regions. This may be attributed to the strategic elevation of the integrated development of the Yangtze River Delta (YRD) region to a national priority by General Secretary Xi Jinping. Anhui Province, leveraging its geographical advantages, has actively integrated into the broader economic development of the YRD region, promoting the convergence and upgrading of the digital economy and essential public health services. This integration has led to a consistent annual increase in the coupling coordination degree between the two systems. In contrast, only Sichuan Province, located in the western region, has reached the “primary coordination” stage, while most other regions remain in a state of dissonance. When essential public health services are relatively stable, a weak digital economy foundation, coupled with insufficient innovation and resource shortages, can hinder the further development of essential public health services, thereby exacerbating regional disparities.

In the long term, an imbalance in the development of either system is not conducive to their coordinated progression. As such, achieving sustained and lasting coupling and coordination between the digital economy and essential public health services requires regions to pursue deeper and broader integration efforts (58). A certain degree of “friction” in the integration process will be necessary to establish a long-term, robust coordination mechanism between these two systems.

### 4.2 Path analysis of coupled and coordinated development of essential public health services and digital economy

#### 4.2.1 Data calibration

Considering the differences in values and units of different variables, calibration processing of variables is required to ensure the consistency and explanatory power of the data. Specifically, in this paper, based on previous experience and research, the three calibration anchor points of full affiliation, crossover point and full non-affiliation were set to 75, 50, and 25% using the direct calibration method, and the raw data were calibrated to fuzzy sets with an interval of 0–1 (63). The calibration anchor points for each variable are shown in Table 4.

**Table 4:** Fuzzy set calibration of variables.

Condition variables	Calibration anchor point		
	Completely affiliated	Intersection	Completely unaffiliated
Coupling coordination	0.719	0.614	0.474
Institutional environment	3.821	3.018	1.501
Government support	9.042	8.302	7.245
Human capital level	177,791.500	130,139.500	90,230.750

#### 4.2.2 Necessary condition analysis

The use of fuzzy set qualitative analysis requires the necessity analysis operation to be carried out on all condition variables to determine whether a condition is necessary for a result to occur (66). Therefore, the calibrated fuzzy values need to be analyzed for necessary conditions (Table 5), and the results show that the consistency of the six condition variables is lower than 0.9, which shows that different conditions will have different impacts on the coupling and coordination of the two systems through linkage matching.

**Table 5:** Necessity analysis results of antecedent conditions.

Condition variables	High coupling coordination		~High coupling coordination	
	Consistency	Coverage	Consistency	Coverage
Institutional environment	0.784	0.845	0.365	0.376
~Institutional environment	0.425	0.393	0.847	0.796
Government support	0.832	0.847	0.261	0.260
~Government support	0.264	0.275	0.846	0.841
Human capital level	0.758	0.742	0.383	0.366
~Human capital level	0.351	0.357	0.735	0.763

#### 4.2.3 Sufficiency analysis of conditional groupings

When performing fuzzy set grouping analysis, it is necessary to test the sufficiency of different combinations of condition variables on the results. Therefore, this paper adopts the test method used by Fiss, setting the case frequency threshold to 1 and the original consistency threshold to 0.80 (67); at the same time, combining with relevant research, the consistency threshold of PRI is set at 0.7 to achieve the purpose of reducing contradictory groupings (57). The fsQCA3.0 software was used for normalization and the results are shown in Table 6.

**Table 6:** High-level configuration results.

Condition variables	Government-led and human capital-driven	Government and institutional environment-driven	Government and human capital-driven
	H1	H2	H3
Institutional environment	⨂	●	
Government support	●	●	●
Human capital level	●	⨂	●
Consistency	0.857	0.805	0.912
Coverage	0.318	0.170	0.596
Unique coverage	0.064	0.104	0.334
Consistency of solutions	0.902		
Coverage of solutions	0.771		

● Represents the existence of core conditions; ● represents the existence of marginal conditions; ⨂ represents the lack of core conditions; ⨂ represents the lack of marginal conditions; blank indicates that the condition has no effect on the result.

##### 4.2.3.1 Government-led and human capital-driven path (H1)

In path H1, government support takes a dominant role, with human capital serving as a secondary driver, while the institutional environment is a marginal, often absent condition. This path demonstrates a characteristic dominant relationship, where government intervention positively influences development but may also exert a crowding-out effect on the market environment (68). Path H1 suggests that even in regions with suboptimal institutional environments, higher levels of government support and human capital investment can still foster coordinated development between the digital economy and essential public health services to a certain extent. In Tianjin, for example, the Tianjin municipal government has paid special attention to building institutional mechanisms for the digital economy, promoting key demonstration projects in the big data industry and the construction of digital infrastructure and actively promoting the improvement of the digital industry chain. Such as digital healthcare, smart city, big data center and smart city projects. Tianjin has also set up a special leading group for the development of digital economy and strengthened cooperation with enterprises and universities to promote the development of digital economy.

##### 4.2.3.2 Government and institutional environment-driven path (H2)

In path H2, the institutional environment is the core driver, with government support acting as a marginal, yet present, condition, while human capital investment is largely absent. This indicates that even when human capital is insufficient, a robust institutional environment, complemented by strong government support, can effectively promote the coordinated development of the digital economy and essential public health services. Gansu Province, for example, is located in western China and has long faced challenges such as relatively poor information infrastructure and a lack of digitized talent. However, in recent years, Gansu Province has gradually accelerated the development of the digital economy through a series of policies and government-driven initiatives. In 2016, it released the “Gansu Province Big Data Industry Development Plan” and set up the “Big Data Development Leading Group” to actively promote the development of the digital economy. In 2018, Gansu Province issued the “Three-Year Action Plan for the Development of Big Data Industry in Gansu Province (2018–2020)” and set up special funds to promote the development of big data industry. Driven by the government, Gansu Province is actively promoting the application of digital health while vigorously developing digital infrastructure.

##### 4.2.3.3 Government and human capital-driven path (H3)

In path H3, both government support and human capital are central conditions, while the institutional environment remains uncertain. This suggests a mutually beneficial relationship between government support and human capital, where the synergy between these two factors creates a reinforcing effect. This symbiosis generates a high level of coordinated innovation, allowing the digital economy and essential public health services to advance in tandem. In Anhui Province, for example, the province is vigorously developing the big data industry, artificial intelligence, cloud computing and other fields, focusing on the development of artificial intelligence and big data industry agglomeration. Hefei, as one of the demonstration cities for digital economy development in Anhui Province, has piloted the “Internet + Healthcare” program. Through the construction of online medical platforms and telemedicine services, it not only improves the efficiency of health resources utilization, but also allows residents, especially the older adult, the disabled and other special groups, to enjoy health services more conveniently. In addition, the Anhui provincial government has increased its efforts to introduce high-level talents in the field of information technology through the “Talent Strengthening Province” program. Through the province’s universities (such as the University of Science and Technology of China), a large number of high-quality talents have been trained, alleviating some of the talent bottlenecks.

A comparison of these three paths reveals a substitution effect between institutional environment and human capital, where either factor, combined with strong government support, can lead to the coordinated development of the digital economy and essential public health services. Notably, government support emerges as a crucial driver across all paths, underscoring the importance of financial and policy backing for achieving high-quality, coordinated development of both systems. Additionally, the consistency in outcomes across different paths highlights the role of the substitution effect. For instance, in comparison to paths H2 and H3, the human capital advantages observed in H3 can substitute for a robust institutional environment. Therefore, regions should adapt their development strategies based on their specific conditions and the evolving development landscape.

#### 4.2.4 Robustness test

To ensure the accuracy of the fuzzy-set qualitative comparative analysis results, a robustness test was conducted to assess the sensitivity of the empirical findings (69). First, the PRI consistency was improved from 0.70 to 0.75, producing essentially identical groupings. Second, this involved modifying the original consistency threshold from 0.80 to 0.85 (70, 71). After adjusting the threshold, the resulting histograms were consistent with those in Table 6, affirming that the findings pass the robustness test.

## 5 Conclusions and policy implications

### 5.1 Conclusion

This study employs a coupling coordination degree model to assess the level of integration and coordination between the digital economy and essential public health services across 30 provinces (including municipalities and autonomous regions) in China. Grounded in the WSR (Wuli-Shili-Renli) methodology, this study utilizes the fuzzy-set qualitative comparative analysis (fsQCA) method to explore the interactive mechanisms and driving pathways through which the institutional environment, government support, and human capital levels influence the coupling and coordinated development of public health services and the digital economy. By identifying the core conditions and intricate relationships shaping the coordination between these two systems, the study yields the following key conclusions:

First, the study reveals that although the coupling coordination degree between the digital economy and essential public health services in China exhibited an upward trend from 2012 to 2021, the overall level remained relatively low, with pronounced regional disparities. The national average coupling coordination degree increased from 0.41 in 2012 to 0.43 in 2021, reflecting an annual growth rate of merely 0.56%, indicating that the synergistic interaction between the two systems requires further enhancement. At the regional level, distinct hierarchical differences are observed across the eastern, central, and western regions, with their respective average coupling coordination degrees reaching 0.57, 0.36, and 0.34. The eastern region demonstrates the highest level of coordination, maintaining a status of “barely coordinated,” suggesting that the digital economy has played a significant role in advancing public health services. However, growth has decelerated, with an annual average increase of only 0.61% and a total increase of 5.45%. In certain provinces, such as Beijing (−1.2%) and Guangdong (−6.3%), the coupling coordination degree has declined, potentially due to the rapid expansion of the digital economy while the public health system remains relatively mature, leaving limited room for further marginal improvements. The central region exhibits the most substantial progress, with an annual growth rate of 3.24% and a total increase of 29.18%, highlighting the potential of the digital economy in enhancing healthcare accessibility. This trend is particularly evident in provinces such as Henan (+81%) and Anhui (+230%), where the integration of digital technologies and policy interventions has yielded significant improvements. Nevertheless, the overall coupling coordination degree in the central region remains below the national average, categorizing it within the “maladaptive decline” stage. Meanwhile, the western region, despite achieving an annual growth rate of 1.85% and a total increase of 16.63%, continues to lag behind the central region and exhibits considerable internal fluctuations. For instance, Qinghai (−11.6%) and Xinjiang (−15.1%) experienced a decline in their coupling coordination degree, likely due to constraints such as inadequate medical resource supply and underdeveloped digital infrastructure. These findings underscore the persistent challenges faced by the western region in integrating digital economic advancements with public health services.

Second, at the urban level, the coupling coordination degree exhibits an increasing trend of spatial divergence, with some cities having entered the coordinated development stage, while the majority remain at a low level of coordination. The study reveals that between 2012 and 2021, only six provincial-level regions, including Beijing, Shanghai, and Jiangsu, progressed to the “coordinated development” stage. In contrast, the coupling coordination degree of most cities declined between 2019 and 2021, highlighting a pronounced spatial differentiation trend. For instance, Anhui Province demonstrated a significantly greater improvement in its coupling coordination degree compared to other regions, likely attributable to the integration strategies of the Yangtze River Delta, which have continuously enhanced the synergistic development of its digital economy and public health services. By comparison, in the western region, only Sichuan Province reached the “primary coordination” stage, while the remaining provinces remained in a state of “maladaptation.” This disparity reflects the constraints imposed by underdeveloped infrastructure, insufficient digital technology innovation, and limited public health resource allocation, which have collectively hindered balanced regional development to a certain extent. From the perspective of regional heterogeneity, the eastern region exhibits greater resilience. For example, in provinces such as Jiangsu and Fujian, the fluctuation in coordination degree remained below 1%, suggesting that their digital economy and public health systems possess strong risk resistance, enabling them to maintain a relatively stable coupling coordination level even in the face of pandemic shocks. In contrast, the central region demonstrates a clear catching-up effect. Provinces such as Hubei and Henan have experienced a sustained increase in coordination degree following the pandemic, reflecting the continuous investment and optimization of digital health infrastructure under the “Rise of Central China” strategy, which has facilitated a more effective alignment between the public health system and digital economic development. However, the western region exhibits significant fluctuations in coordination degree. For instance, Xinjiang experienced pronounced oscillations in 2017 (0.38), 2019 (0.43), and 2021 (0.45), indicating that its digital epidemic prevention system remains in an unstable state. This instability may be influenced by factors such as the completeness of infrastructure, policy implementation efficiency, and regional disparities in resource allocation. Therefore, it is essential for different regions to further optimize their coordination mechanisms and adopt differentiated strategies tailored to their respective development stages. This approach can help prevent structural imbalances caused by either premature or lagging system development, ultimately facilitating the high-quality coordinated development of the digital economy and public health services.

Third, government support is the core driving force behind the coupling and coordinated development of the digital economy and public health services, while institutional environment and human capital exhibit a certain degree of substitution effect across different developmental pathways. The study indicates that across the three identified developmental pathways (H1, H2, and H3), government support consistently serves as the primary driving factor, underscoring the critical role of financial investment, policy support, and governmental guidance in fostering the integration of the two systems. However, institutional environment and human capital levels demonstrate a degree of substitution in different developmental pathways. For instance, in the H1 pathway, despite a weak institutional environment, strong government support combined with high human capital investment can still promote coupling coordination. In the H2 pathway, even in the presence of low human capital levels, a well-established institutional environment and effective policy guidance can still facilitate the integration of the two systems. The H3 pathway further reveals the synergistic effect between government support and human capital, illustrating that their mutual reinforcement can lead to a high-level coordinated innovation model. Therefore, while government-led initiatives remain fundamental, different regions should flexibly adjust their institutional environment development or human capital investment based on their unique resource endowments to identify the optimal developmental pathway.

Fourth, the configuration of the institutional environment and human capital determines the level of coupling and coordinated development across different regions. While all three developmental pathways highlight the central role of government intervention, the choice of pathway varies based on regional conditions. For example, Tianjin, Gansu, and Anhui have all successfully achieved integration between the digital economy and public health services, yet each has followed a distinct pathway with unique underlying mechanisms. Tianjin (H1 pathway) has relied on government-driven initiatives and substantial human capital investment to advance digital healthcare, even in the presence of a relatively weak institutional environment. Gansu (H2 pathway), by contrast, has leveraged a well-established institutional framework to accelerate the adoption of digital health applications through policy guidance, despite its relatively limited human capital resources. Meanwhile, Anhui (H3 pathway) has fostered a highly efficient digital economy development model through the synergistic effect of strong government support and a high level of human capital, driving the deep integration of digital technologies with public health services. These findings suggest that while government support remains the core driving force, the rational allocation of the institutional environment and human capital plays a decisive role in shaping regional developmental pathways and determining the level of coupling coordination. Therefore, each region should tailor its development model based on its specific conditions, taking into account government policies, institutional frameworks, and human capital resources to optimize its approach and enhance the coordinated development of the digital economy and public health services.

#### 5.1.1 Urban-level trends and spatial disparities

At the urban level, the coupling coordination degree exhibits an increasing spatial divergence. While some cities have entered the coordinated development stage, the majority remain at a low coordination level. From 2012 to 2021, only six provincial-level regions, including Beijing, Shanghai, and Jiangsu, reached the “coordinated development” stage. In contrast, the coupling coordination degree declined in most cities between 2019 and 2021, exacerbating spatial disparities. For instance, Anhui Province demonstrated the most significant improvement, likely due to the Yangtze River Delta integration strategy, which has facilitated the alignment of digital economy advancements with public health services. Conversely, the western region has struggled to keep pace, with only Sichuan Province achieving a “primary coordination” level, while other provinces remain in a “maladaptive” state. This suggests that weak infrastructure, insufficient digital technology innovation, and constrained public health resource allocation continue to hinder regional balance. From a regional heterogeneity perspective: Eastern Region: Demonstrates strong resilience, with provinces such as Jiangsu and Fujian maintaining fluctuations of less than 1%, indicating robust risk resistance and system stability, even under external shocks such as pandemics. Central Region: Exhibits a catch-up effect, with provinces such as Hubei and Henan experiencing sustained increases in their coordination levels post-pandemic. This reflects the influence of the “Rise of Central China” strategy, which has facilitated continuous investments in digital health infrastructure, enhancing the adaptability of the public health system to digital economic developments. Western Region: Displays considerable volatility. For instance, Xinjiang’s coupling coordination degree fluctuated sharply between 2017 (0.38), 2019 (0.43), and 2021 (0.45), indicating that the development of digital epidemic prevention systems remains unstable. This instability may be attributed to variations in infrastructure quality, policy implementation efficiency, and regional resource allocation. To achieve high-quality coordination between the digital economy and public health services, regions should refine their collaborative mechanisms and adopt differentiated strategies tailored to their developmental stages. Such an approach would help mitigate structural imbalances arising from the disproportionate advancement of these systems.

#### 5.1.2 Government support as the core driver, with institutional and human capital exhibiting substitutive effects across pathways

The study identifies government support as the primary driving force behind the coupling and coordinated development of the digital economy and public health services, while the institutional environment and human capital demonstrate substitutive effects across different pathways. Across the three identified development pathways (H1, H2, and H3), government support consistently emerges as the critical driver, underscoring the pivotal role of fiscal investment, policy frameworks, and governmental guidance in fostering system integration. However, institutional environment and human capital display a substitutive relationship: H1 Pathway: Strong government support and high human capital investment can promote coupling coordination, even in the absence of a well-established institutional environment. H2 Pathway: A robust institutional environment, combined with policy guidance, can effectively drive system integration even when human capital levels are low. H3 Pathway: The synergistic effect of government support and human capital fosters a high-level collaborative innovation model, wherein these factors reinforce one another. Thus, regional governments should leverage their unique resource endowments, dynamically adjust institutional development or human capital investments, and identify optimal development pathways based on local conditions.

#### 5.1.3 Institutional environment and human capital configuration as determinants of regional development pathways

The allocation of institutional environment and human capital significantly influences the level of coupling and coordination across different regions. While government support remains the fundamental driver, the selection of development pathways should be tailored to regional characteristics. For example: Tianjin (H1 Pathway): Relied on government-driven initiatives and human capital investments, successfully advancing digital healthcare despite a relatively weak institutional environment. Gansu (H2 Pathway): Leveraged a well-established institutional environment to compensate for limited human capital resources, accelerating digital health adoption through policy interventions. Anhui (H3 Pathway): Achieved deep integration of digital technology and public health services by capitalizing on strong government support and high human capital levels, forming an efficient digital economy development model. These findings underscore the necessity for regional policymakers to adopt strategic optimization approaches, considering the interplay between institutional capacity, human capital resources, and policy frameworks. By tailoring their development models to local conditions, regions can effectively enhance the coordinated development of the digital economy and public health services.

Although this study aims to reveal the level of coupled coordination and optimization path of digital economy and essential public health services, it still has some limitations. Firstly, this study analyzed the level of coupled coordination in each region at the macro level, which can be further explored at the micro level in the future. Second, the indicators calculated through the entropy method may not be completely objective in the assignment process. In the future, a better evaluation system for the indicators will be established, combining expert ratings and field studies to further enhance their significance as a guide for public health.

### 5.2 Policy implications

#### 5.2.1 Accelerating digital economy development to achieve a high-level coupling and coordinated development of both systems

At present, significant disparities persist in the coupling and coordination levels between the digital economy and essential public health services across different provinces and cities in China. Therefore, governments at all levels should actively facilitate the integration of digital technologies into the healthcare sector and strengthen the training of digital health professionals. In particular, optimizing and expanding infrastructure in areas such as digital health and smart healthcare is crucial to ensuring the steady advancement of both the digital economy and essential public health services. Furthermore, healthcare institutions across regions should effectively leverage digital technologies to enhance the allocation of healthcare resources, promoting the intelligent, personalized, and precise development of medical services. This approach aims to ensure the accessibility and universality of essential public health services. Finally, the government can establish appropriate incentive mechanisms to encourage healthcare institutions at all levels to undergo digital transformation. By doing so, the intelligent development of the healthcare sector can be further promoted, ultimately facilitating the efficient coupling of the digital economy with essential public health services.

#### 5.2.2 Emphasizing regional synergies and leveraging the demonstration effect of high coupling and coordination provinces

The coupling and coordinated development of the digital economy and essential public health services exhibit significant spatial interdependencies, necessitating that policymakers fully consider the synergetic effects across different regions. On the one hand, regions with high levels of coupling and coordination should actively serve as role models, fostering a “prosperous neighbors” effect by promoting industrial relocation, technology spillovers, and targeted assistance programs to support the development of less-coordinated areas. On the other hand, achieving an efficient coupling between the digital economy and essential public health services relies on high-quality resources and cross-sector collaboration. By implementing a “co-construction and sharing” approach to infrastructure, administrative barriers between regions can be mitigated, thereby accelerating the flow and diffusion of knowledge, technology, and capital. This, in turn, helps to narrow regional disparities in coupling coordination and enhances the overall level of integrated development. Furthermore, governments can facilitate industry-academia-research collaboration to establish a bridge between essential public health services, the digital economy, and technological innovation. By fostering interdisciplinary and interregional cooperation, these efforts can drive the digital transformation of essential public health services.

#### 5.2.3 Strengthening multi-factor synergies and enhancing the leading role of Core driving factors

The efficient coupling and coordination between the digital economy and essential public health services result from the interplay of multiple factors, with varying driving pathways across different regions. Therefore, in promoting their coordinated development, it is crucial to adopt region-specific approaches tailored to local conditions. On the one hand, the synergy between technological, organizational, and environmental factors should be strengthened. Development strategies should be formulated based on regional resource endowments, economic development levels, innovation capabilities, and human capital availability to explore locally adaptive pathways. For instance, in regions with relatively low levels of human capital but a strong industrial foundation, coupling coordination can be enhanced through technological innovation and industrial structure optimization. On the other hand, the government should continuously increase investment in key technological research and development, particularly in digital technologies closely related to essential public health services. Advancements in critical areas such as telemedicine and digital healthcare services should be promoted to further enhance the coupling and coordination between the digital economy and essential public health services.

## Data Availability

Publicly available datasets were analyzed in this study. This data can be found at: www.resset.com.
